# Icariin-Loaded Hydrogel Regulates Bone Marrow Mesenchymal Stem Cell Chondrogenic Differentiation and Promotes Cartilage Repair in Osteoarthritis

**DOI:** 10.3389/fbioe.2022.755260

**Published:** 2022-02-09

**Authors:** Yuefeng Zhu, Le Ye, Xiaoxi Cai, Zuhao Li, Yongqian Fan, Fengjian Yang

**Affiliations:** ^1^ Department of Orthopedics, Huadong Hospital Affiliated to Fudan University, Shanghai, China; ^2^ Department of Pain, Renji Hospital, Shanghai Jiaotong University School of Medicine, Shanghai, China

**Keywords:** icariin, hydrogel, BMSCs, osteoarthritis, cartilage repair, pain relief

## Abstract

Intra-articular injection of mesenchymal stem cells is a potential therapeutic strategy for cartilage protection and symptom relief for osteoarthritis (OA). However, controlling chondrogenesis of the implanted cells in the articular cavity remains a challenge. In this study, hydrogels containing different concentrations of icariin were prepared by *in situ* crosslinking of hyaluronic acid and Poloxamer 407. This injectable and thermoresponsive hydrogel, as a 3D cell culture system, showed good biocompatibility with chondrocytes and bone marrow mesenchymal stem cells (BMSCs), as well as promoted proliferation and chondrogenesis of BMSCs through the Wnt/*β*-catenin signaling pathway. Intra-articular injection of this kind of BMSC-loaded composite hydrogel can significantly prevent cartilage destruction by inducing chondrogenic differentiation of BMSCs, and relieve pain through regulating the expression of inflammatory cytokines (e.g., IL-10 and MMP-13) in the OA model. Incorporating BMSCs into this novel icariin-loaded hydrogel indicates a more superior efficacy than the single BMSC injection, which suggests a great potential for its application in OA.

## Introduction

Osteoarthritis (OA) is a chronic bone and joint disease characterized by articular cartilage degeneration and destruction, as well as hyperosteogeny in weight-bearing areas. Up to now, OA affects 303.1 million people worldwide and is mainly manifested as joint pain and limited mobility, which seriously limit the limb function and quality of life (Peat and Thomas, 2021). Due to the restricted ability of articular cartilage regeneration in adults, articular cartilage degeneration is considered as an irreversible pathophysiological change. The existing conservative treatment strategies, such as taking nonsteroidal anti-inflammatory drugs, intra-articularly injecting glucocorticoid and hyaluronic acid, etc., have not achieved satisfactory outcomes in alleviating OA, and can only temporarily alleviate the disease progression. Arthroplasty is considered to be an effective method for the treatment of end-stage OA, while it often brings heavy economic burden, long recovery time, and some serious complications including infection, prosthesis loosening and displacement, periprosthetic fractures, and so on (Bai et al., 2020b; Zhao et al., 2020). Therefore, it is of great significance to develop early nonsurgical treatment to prevent the progression of OA.

With the rapid development of regenerative medicine, the application of stem cells and cytokines has gradually become a novel strategy for the remedy of OA. Among the stem cells, mesenchymal stem cells (MSCs), especially bone marrow mesenchymal stem cells (BMSCs), are regarded as important seed cells for the treatment of OA due to their advantages of easy acquisition and extensive sources, advanced proliferative ability, significant multidirectional differentiation potential, and the capacity to regulate inflammation (Barry and Murphy, 2013; Bertoni et al., 2021; Hwang et al., 2021). Various clinical studies conducted by a similar technique revealed that intra-articular injection of MSCs was helpful to ameliorate cartilage destruction and relieve pain in OA (Centeno et al., 2008; Csaki et al., 2008; Maumus et al., 2011). However, after intra-articular administration of MSCs, only a few cells adhered to the cartilage defects, and their differentiation ability was not prominent in the joint cavity (Koga et al., 2008). In addition, the chondrogenic differentiation of MSCs after intra-articular injection was not well controlled, which might limit their clinical applications (Chang et al., 2016; Ha et al., 2019). Therefore, MSCs with more chondrogenic differentiation potential will contribute to the efficient repair of articular cartilage in OA.

Hydrogel is a three-dimensional (3D) network composed of hydrophilic polymers, which sustains similar physical and chemical properties as the extracellular matrix (ECM) (Liu et al., 2018; Liu et al., 2021). As an ideal 3D cell culture system, hydrogels with specific properties are capable of inducing chondrogenic differentiation of MSCs (Yao et al., 2017; Li et al., 2020; Li et al., 2021a). For instance, adding bioactive ingredients in the hydrogels, *Epimedium*, a traditional Chinese medicine, has been widely studied in anti-osteoporosis and alleviation of OA (8). Icariin, whose chemical formula is C_33_H_40_O_15_, is the primary pharmacologically active component of the Chinese herb medicine *Epimedium*. Previous studies have indicated that icariin can accelerate cartilage ECM synthesis and inhibit ECM degradation, upregulate the cartilage-specific gene expression of chondrocytes, and induce directed chondrogenesis of BMSCs without hypertrophic differentiation (Sun et al., 2013; Wang et al., 2014; Wang et al., 2018). Greatly inspired by previous studies, icariin encapsulated in hyaluronic acid/collagen hydrogel can maintain biological activity and plays a good role in osteogenesis and cartilage induction (Yang et al., 2018; Liu et al., 2019). Consequently, icariin may have potential applications in cartilage tissue engineering.

**SCHEME 1:**
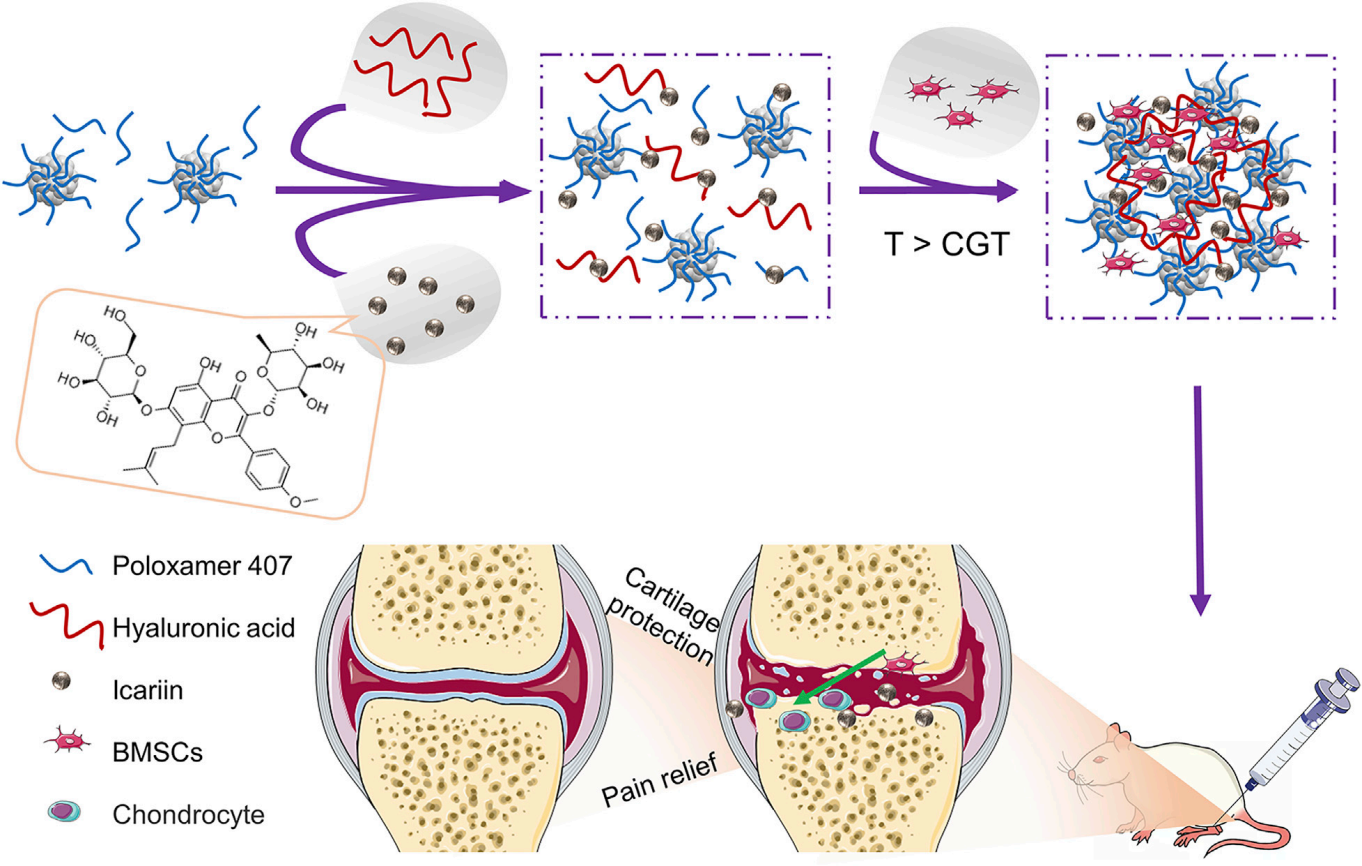
Schematic illustration of BMSCs incorporating icariin-Poloxamer 407 and HA (PHa) hydrogels for cartilage protection and pain relief in osteoarthritis (OA).

In this study, an injectable and thermoresponsive hydrogel containing different concentrations of icariin were prepared by *in situ* crosslinking of hyaluronic acid and Poloxamer 407. Then the hydrogel containing the optimal concentration of icariin was applied to encapsulate BMSCs for intra-articular injection in OA rats. As a result, in hydrogels containing the optimal concentration of icariin, BMSCs can fully differentiate into chondrogenic, inhibit inflammation, and show a preferable outcome on cartilage destruction and pain relief in OA (Scheme 1). As far as we know, it is the first attempt to explore the synergistic action of BMSCs and icariin-loaded thermoresponsive hydrogel for intra-articular injection in OA.

## Materials and Methods

### Materials

Poloxamer 407 (P407, culture tested), fluorescein isothiocyanate-conjugated goat anti-mouse IgG, and etramethylrhodamine isothiocyanate-conjugated goat anti-rabbit IgG were provided by Sigma-Aldrich (Gillingham, UK). Hyaluronic acid (HA), ∼1,000 kDa, was obtained from Bioland Co., Ltd. (Cheonan, Korea). Icariin (purity: 99%) was purchased from the National Institute for the Control of Pharmaceutical and Biological Products (Beijing, China). Low-glucose Dulbecco’s modified Eagle’s medium (LG-DMEM), streptomycin–penicillin, and fetal bovine serum (FBS) were obtained from Gibco (Grand Island, NY, United States). Chondro-inductive medium (catalog no. GUXMX-90041) was obtained from Cyagen (Guangzhou, China), and TGF-*β*3 was supplied by PeproTech (Cranbury, NJ, United States). Rat BMSCs (catalog no. CP-R131) and chondrocytes (catalog no. CP-R087) were provided by Procell (Wuhan, China). Cell Counting Kit-8 (CCK-8) and Calcein acetoxymethyl ester (Calcein-AM)/Propidium Iodide (PI), BCA Protein Assay Kit, and RIPA Lysis Buffer were supplied by Beyotime Biotechnology (Shanghai, China). The 4% paraformaldehyde solution, phosphate-buffered saline (PBS), and Triton X-100 were provided by Beijing Solarbio Science and Technology (Beijing, China). Eastep Super Total RNA Extraction Kit was purchased from Promega Corporation (Shanghai, China). Perfect Real Time RT reagent kit, Prime Script RT reagent kit, and SYBR Premix Ex Taq II kit were obtained from Takara Biotechnology (Dalian, China). TRIzol Reagent was supplied by Invitrogen Life Technology (Carlsbad, CA, United States). Safranin O, Alcian blue, hematoxylin eosin (H&E), safranin O-fast green stains, enhanced chemiluminescence reagent, and 4′,6-diamidino-2-phenylindole 2HCl (DAPI) were obtained from Thermo Fisher Scientific (Shanghai, China). Primary antibodies, including Sox 9, Col-2a1, Aggrecan, HIF-1*α*, Dvl1, Gsk3*β*, *β*-catenin, IL-10, and MMP-13 were provided by Abcam (Cambridge, United Kingdom), and secondary antibodies were provided by Jackson ImmunoResearch Laboratories (West Grove, PA, United States).

### Icariin-Loaded Hydrogel Preparation and Characterization

A simple mixing process is applied to prepare the Poloxamer 407 and HA (PHa) hydrogel in aqueous solution according to previous studies (Jung et al., 2017; Hsieh et al., 2020). In brief, Poloxamer 407 (20 wt%) and HA (1 wt%) were dispersed in double distilled water for a further 24 h in a refrigerator at 4°C until a transparent solution was obtained. Then various amounts (0, .2, 1.0, and 5.0 mg) of icariin were dispersed into every 10 ml of PHa solution for dissolution with gentle stirring for 1 h, that is, the concentrations of icariin in terms of molarity were 0, 29.6, 147.8, and 738.9 μM. Finally, hydrogels containing different concentrations of icariin (abbreviated as PHa, PHa@I-L, PHa@I-M, and PHa@I-H, respectively, according to the icariin concentration) were obtained and stored at 4°C for subsequent investigations.

The PHa solution was subsequently warmed to 37°C to change the PHa from solution into gel. To detect the temperature of the PHa hydrogel to sol–gel transition, rheological measurement was carried out from 0°C to 40°C through a rheometer (Malvern, UK). In addition, the microscopic structure of the hydrogel was detected by a scanning electron microscope (SEM, JSM-6700F, JEOL, Japan) after freeze drying. For degradation detection, the PHa hydrogels were incubated in 1 ml of PBS with or without 100 μg ml^−1^ of hyaluronidase at 37°C. At preset time points, the hydrogels were taken out, and their wet weights (W_t_) were weighed and recorded. The degradation rate was reparented by following the equation: degradation rate = (W_0_−W_t_)/W_0_ × 100%, where W_0_ is the initial wet weight of the sample. To investigate the release profiles of icariin from the hydrogels, 1 ml of icariin-loaded hydrogels were immersed in 10 ml of PBS at 37°C. At predetermined time points, the PBS was collected and replaced by 10 ml of fresh PBS. The concentrations of icariin in the samples were determined by UV spectrophotometry. Absorbances of the standard solution and samples were measured at the icariin maximum absorption wavelength of 270 nm. The cumulative release of icariin was quantified according to the standard curve of icariin.

### Cell Proliferation and Viability

To evaluate the biocompatibility of 3D network composed by PHa hydrogel with different concentrations of icariin to BMSCs and chondrocytes, the proliferation and survival of cells encapsulated in hydrogels were detected by CCK-8 and Calcein-AM/PI. Briefly, BMSCs and chondrocytes were seeded into the plates (abbreviated as NC group), PHa, PHa@I-L, PHa@I-M, and PHa@I-H hydrogels, respectively. In the NC group, cells were seeded at a density of 2 × 10^4^/well in 24-well plates. In the hydrogel groups, cells were encapsulated in 400 μl of hydrogels as the 3D cell culture system. Specifically, 2 × 10^4^ cells were added into different gel solutions (400 μl) by pipetting evenly at 15°C at a density of 5 × 10^4^ cells/cm^3^. Then the samples were injected into the cell culture plate and transferred into the incubator at 37°C for gelation immediately. Each well was added 1 ml of medium for cell culturing in a humidified environment with 37°C and 5% CO_2_. The culture medium was replaced every 3 days. At the scheduled time point, the proliferation of cells was studied by a CCK-8 assay. In brief, after changing the medium, 10% CCK-8 solution was added to each well and incubated for 2 h at 37°C. Finally, 100 μl of solution of each sample was transferred into a 96-well plate and measured by a microplate reader (Varioskan LUX, Thermo Scientific) at 450 nm. In addition, Calcein-AM/PI staining was conducted after 3 days of incubation according to the directions. Briefly, 2 μM Calcein-AM and 4.5 μM PI were added into the samples, and then incubated for 15 min at 37°C in the dark. The cell viability was observed and recorded by an Olympus FV1000 confocal laser scanning microscope (CLSM), and the cell survival rate was analyzed by ImageJ software (1.50i version, National Institutes of Health) according to the proportion of green-stained living cells in the total number of cells.

### Safranine O and Alcian Blue Staining

For chondrogenic differentiation induction, BMSCs in the different groups were induced by TGF-*β*3 containing chondrogenic induction medium for 12 days. Then the samples were stained with safranin O and Alcian blue in accordance with the instructions of the manufacturer. In brief, after being fixed with 4% paraformaldehyde for 5 min, the samples were stained with safranin O for 5 min, and another batch of samples was stained by Alcian blue for 30 min at room temperature. Then the images were observed by an optical microscope (DSX 500, Olympus, Japan).

### Real-Time Quantitative PCR

To evaluate the gene expression of *Sox 9*, *Col-2a1*, *Aggrecan*, *HIF-1α*, *Dvl1*, *Gsk3β*, *β-catenin*, *IL-10*, and *MMP-13* in the BMSCs and/or cartilage tissue, RT-qPCR was performed. Total RNA was extracted with TRIzol reagent, and cDNA was synthesized in reverse transcription reaction by 1 µg of total RNA using a Prime Script RT reagent kit. The expression of target genes was detected by qPCR using the SYBR Premix Ex Taq II kit in accordance with the instructions of the manufacturer. Amplification was performed in 96-well optical reaction plates on the LightCycler 480 (Roche Diagnostics). The amplification was performed using the following program: 94°C for 3 min to activate polymerase, 40 cycles at 94°C for 20 s, 56°C for 20 s, and 72°C for 20 s; melting curve analysis was conducted after every run by heating up to 95°C to monitor the presence of unspecific products. *GAPDH* was used as internal controls for mRNA expression. Primer sequences of genes are displayed in Table 1. The relative RNA expression was analyzed by the formula of the 2^−ΔΔCT^ method.

**TABLE 1:** Primer sequences of genes.

mRNA	Oligonucleotide primers (5′–3′)	NCBI reference sequence
*GAPDH*	F-ATGGGAAGCTGGTCATCAAC	NCBI Reference Sequence: NM_017008.4
	R-GGATGCAGGGATGATGTTCT	
*Sox 9*	F-AGGAAGCTGGCAGACCAGTA	NCBI Reference Sequence: NM_080403.2
	R-CGGCAGGTATTGGTCAAACT	
*Col-2a1*	F-CGAGGTGACAAAGGAGAAGC	NCBI Reference Sequence: NM_012929.1
	R-CTGGTTGTTCAGCGACTTGA	
*Aggrecan*	F-TGGCATTGAGGACAGCGAAG	NCBI Reference Sequence: XM_039101034.1
	R-TCCAGTGTGTAGCGTGTGGAAATAG	
*HIF-1α*	F-ACAAGTCACCACAGGACAG	NCBI Reference Sequence: NM_024359.2
	R-AGGGAGAAAATCAAGTCG	
*Dvl1*	F-TCACCGACTCTACCATGTCC	NCBI Reference Sequence: NM_031820.1
	R-ATACGATCTCCCGAAGCAC	
*GSK3β*	F-CTGCCCTCTTCAACTTTACC	NCBI Reference Sequence: NM_032080.1
	R-TATTGGTCTGTCCACGGTCT	
*β-catenin*	F-TCTAGTGCAGCTTCTGGGTT	NCBI Reference Sequence: NM_053357.2
	R-GATGGCAGGCTCGGTAATG	

### Western Blotting

The samples were lysed with lysis buffer, and the total protein contents of the lysates were evaluated by the BCA protein assay kit. Subsequently, proteins were fractionated by sodium dodecyl sulfate-polyacrylamide gel electrophoresis (SDS-PAGE) with 5% stacking gels and 10% separating gels, and transferred onto a PVDF membrane. Subsequently, the membranes were blocked with 5% nonfat milk at room temperature for 1 h, and then primary antibodies against Dvl1 (1:500), Gsk3*β* (1:250), and *β*-catenin (1:500) were applied. The bands were visualized by the enhanced chemiluminescence reagent and analyzed by ImageJ software.

### Double Immunofluorescent Staining

Immunofluorescent staining of cells was performed using cultured BMSCs on polylysine-coated glass coverslips under chondrogenic inductive conditions for 12 days. The cells were washed with PBS and then blocked with 2% normal goat serum and 1% BSA, allowed to react with rabbit monoclonal anti-Sox 9 (1:200) and rabbit polyclonal anti-Aggrecan antibody (1:400) overnight at 4°C, and incubated with fluorescein isothiocyanate-conjugated goat anti-mouse IgG (1:50) for 1 h at room temperature. The cells were incubated with either mouse monoclonal anti-Col-2a1 (1:200) or mouse monoclonal anti-HIF-1α antibody (1:500) for 1 h at room temperature, and then incubated with tetramethylrhodamine isothiocyanate-conjugated goat anti-rabbit IgG (1:50) for 1 h at room temperature. For counterstaining, the nuclei were stained with DAPI for 5 min at room temperature. The double immunofluorescence-stained samples were observed by a CLSM. In addition, the cartilage samples from *in vivo* experiments were fixed in 4% paraformaldehyde, and then decalcified with .5 M EDTA solution for 4 weeks and embedded for paraffin sectioning to acquire sections about 5 μm. The sections were incubated with primary antibodies against Sox 9, Col-2a1, Aggrecan, HIF-1*α*, Dvl1, Gsk3*β*, *β*-catenin, IL-10, and MMP-13 for immunofluorescence staining in the cartilage.

### Rat Osteoarthritis Model Preparation and Intra-articular Injection

Sprague–Dawley rats (male, 8-week-old) were provided by the Experimental Animal Center of Shanghai Medical College, Fadan University. All animal operations were performed in accordance with the guidelines for Care and Use of Laboratory Animal Experience and approved by the Animal Care and Use Ethics Committee of Huadong Hospital Affiliated to Fudan University.

To prepare the OA rat model, the surgical destabilization of the medial meniscus (DMM) model was carried out by medial collateral ligament transaction under general anesthesia by intraperitoneal injection of 3% pentobarbital at a dose of .2 ml/100 g as described previously (Glasson et al., 2007; Shao et al., 2021). After 2 weeks of DMM procedure, the OA rats were divided into four groups (the number of animals in each group is 10) randomly and then treated with intra-articular injection of different therapeutic agents every 2 weeks three times. The groups were named as follows: 400 μl PBS (abbreviated as PBS Group), 400 μl PHa@I-M hydrogel (abbreviated as PHa@I Group), 2 × 10^6^ BMSCs (abbreviated as BMSCs Group), and 400 μl 2 × 10^6^ BMSC-loaded PHa@I-M hydrogel (abbreviated as PHa@I-BMSCs Group). At 12 weeks after intra-articular injection, the rats were euthanized by an overdose of pentobarbital and the articular cartilage was collected for further detection.

### Histological Evaluation

After scarification, the cartilage samples from OA knee joints were collected. For histological examination, the samples were fixed by 4% paraformaldehyde, and then decalcified with .5 M EDTA solution for about 6 weeks and embedded for paraffin sectioning. Subsequently, the samples were sectioned longitudinally with a thickness of 5 μm. After dewaxing, the slices were stained with H&E and safranin O-fast green according to the standard protocols of the manufacturer. The images of weight-bearing part of the joints were then observed and imaged under a microscope. The Osteoarthritis Research Society International (OARSI) cartilage histopathology assessment system and the Mankin scoring system were applied to assess the destruction of articular cartilage (Kang et al., 2017).

### Behavioral Studies of the Pain Relief

The pain-related behavior of OA rats after intra-articular injection of various therapeutic agents was evaluated by weight-bearing index (WBI) by a 3D gait analysis system (Kinama Tracer, Japan) and paw-withdrawal threshold (PWT) by the von Frey filament (Ugo Basile, Varese, Italy). Briefly, for WBI detection, rats walked along the runway equipped with mechanical sensors, which can record the ground reaction force for each foot, reacting to the weight supported by the corresponding limb. The values were represented by the following formula: WBI = ipsilateral weight/(ipsilateral weight + contralateral weight) × 100%. PWT was conducted to evaluate the efficacy on pain threshold as described previously (Micheli et al., 2019). In addition, PWT, reflecting the mechanical allodynia (hypersensitivity), was investigated according to the force exerted by the von Frey filament ranging from 0 to 40 g with a .2-g accuracy. The paw sensitivity threshold was recorded as the minimum force for leading to a strong and immediate withdrawal reflex of the paw.

### Statistical Analyses

Statistical analysis was conducted by SPSS 20.0 (SPSS Inc., Chicago, IL, United States). Statistical significance comparing two groups with parametric data was evaluated by two-tailed t-test, and multiple groups with parametric data were conducted by one or two-way ANOVA analysis followed by Tukey’s *post-hoc* test. A value of *p* < .05 was regarded as statistically significant between groups. All results were presented as mean ± SD (standard deviation). All experiments in the present study were carried out at least three times independently.

## Result and Discussion

### PHa Hydrogel Preparation and Characterization

**FIGURE 1:**
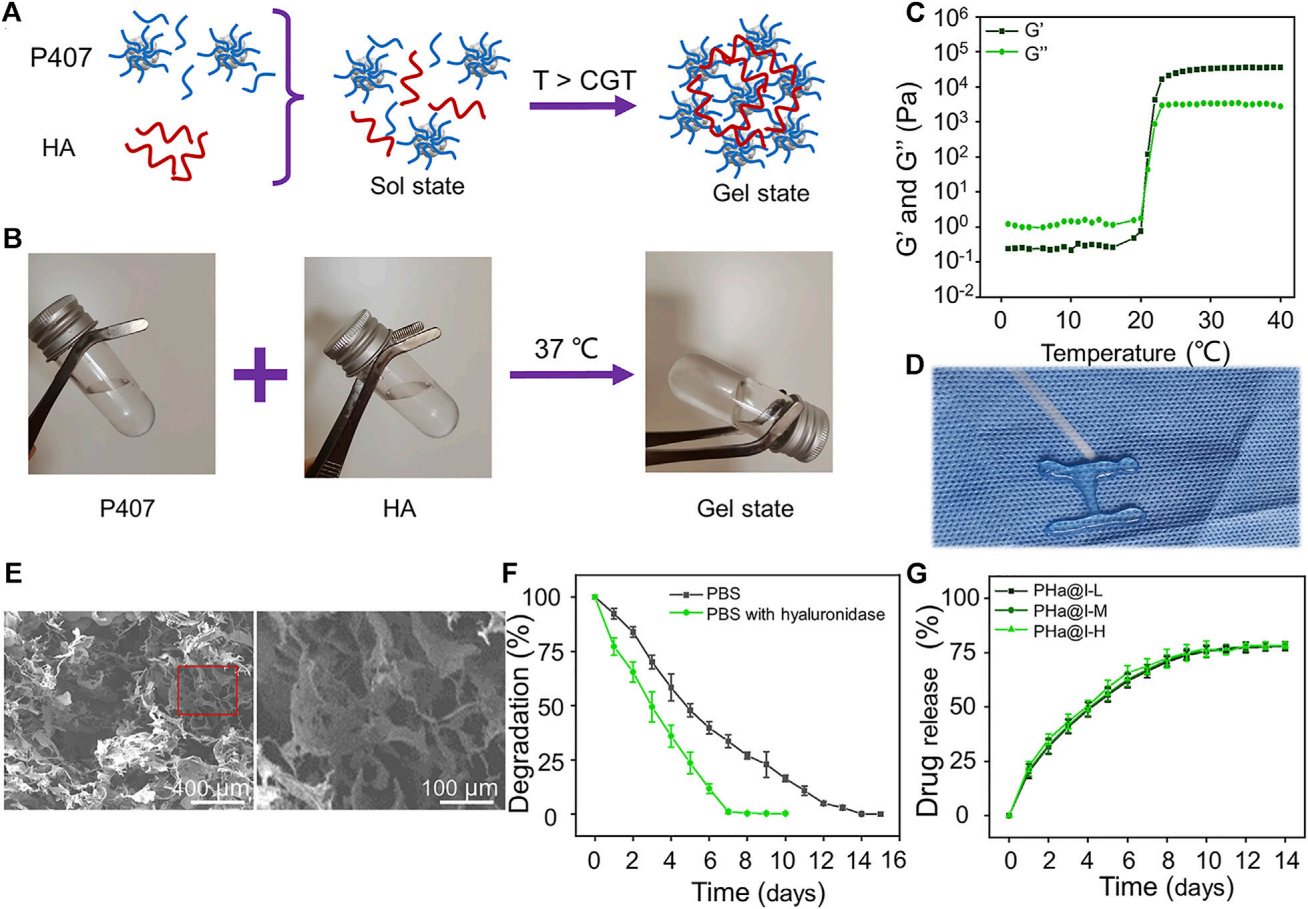
Preparation and characterization of Poloxamer 407 and HA (PHa) hydrogels. **(A)** Schematic illustration of PHa hydrogel preparation. **(B)** The optical images of hydrogel sol–gel transition. **(C)** Storage modulus (*G*′) and loss modulus (*G*″) of hydrogel as a function of temperature. **(D)** The injectable and thermosensitive hydrogel rapidly form gel when injected into a hot plate at 37°C. **(E)** Morphologies of hydrogel observed by SEM. **(F)** Degradation rate of the PHa hydrogels in phosphate-buffered saline (PBS) at 37°C. **(G)** Release profile of icariin from the PHa hydrogels in PBS at 37°C (*n* = 3).

Poloxamer 407, a biocompatible polymer, was utilized widely as a thermosensitive material, owing to its performance of rapid thermo-reversible sol–gel transition and the capability to stabilize sustained releasing drugs (Bai et al., 2020a; Hsieh et al., 2020). Although the surfactant function of Poloxamer 407 is beneficial to the dispersion of drugs in the hydrogels, this capacity may result in the rapid erosion of the hydrogels in several hours under physiological conditions, thus, leading to a burst release of the loaded drugs (Tundisi et al., 2021; Yang et al., 2021). To address these limitations, HA, a biopolymer naturally presenting in the synovial fluid, was employed in this study as a carbohydrate additive for controlled release of drugs to reduce the concentration of Poloxamer 407 required for gelation. In addition, intra-articular administration of HA is regarded as a cost-effective treatment as viscosupplementation to delay cartilage destruction and relieve OA symptoms (Henrotin et al., 2015; He et al., 2017). The addition of high-molecular-weight HA could help the dense packing of Poloxamer 407 micelles at the gelation temperature (i.e., above lower critical solution temperature, Figure 1A). The driving force for this phenomenon could be a hydrophobic interaction between acetyl groups on HA and methyl groups on Poloxamer 407. As a result, the inter-micellar crosslinking induced by HA may heighten the mechanical strength and retention time of the prepared thermosensitive hydrogel (Jung et al., 2017).

As shown in Figure 1B, the PHa solution underwent a sol–gel transition within 60 s by temperature rising from 4°C to 37°C. To determine the critical gelation temperature (CGT) of sol–gel transition, the rheological behavior was detected, and the results indicated that both the storage modulus (*G*′) and loss modulus (*G*″) of the hydrogel were rapidly promoted after 21°C, indicating that the CGT temperature of the PHa hydrogel was ∼21°C (Figure 1C). The most attractive feature of the PHa hydrogel is its reversible thermo-responsive property, allowing it to undergo gelation close to body temperature (>CGT) and remain at the site of implantation as a continuous drug delivery device. This reversible thermo-responsive nature also gives the hydrogel injectable property. As shown in Figure 1D, the PHa solution was extruded through a syringe onto a hot plate (37°C), and it can form into gel state rapidly. SEM observation showed that the hydrogels had an interconnected pore structure with a diameter of about 100 μm (Figure 1E). These porous structures of the hydrogels could promise nutrient and oxygen transportation, enable cellular penetration, migration, and proliferation.

The degradation behaviors of the hydrogels are shown in Figure 1F. The PHa hydrogel in the PBS can be completely degraded within 14 days. However, in the presence of hyaluronidase, the degradation of hydrogels was accelerated and almost degraded on the seventh day. This may be due to the destruction of chemical bonds by hyaluronidase, but the main framework of poloxamer self-assembled hydrogel still existed to prevent instantaneous disruption of the network structure. Hydrogels, as cross-linked polymer networks, have emerged as particularly promising materials for drug delivery. Drugs can be loaded into hydrogels through a manifold of mechanisms and strategies. Here, we speculate that icariin is retained in the hydrogel through the formation of hydrogen bonds and being physically entrapped in hydrogel (Vermonden et al., 2012; Li et al., 2021b; Wang et al., 2021). The release curves of icariin in PHa@I-L, PHa@I-M, and PHa@I-H groups were similar, as illustrated in Figure 1G. The release rate of icariin on the first day was slightly faster, and the release rate gradually decreased in the subsequent days, indicating a controlled and sustained drug-release profiles. On the 12th day, the total proportion of released icariin was up to 77.33 ± 2.39%, 77.44 ± 1.81%, and 78.13 ± 1.00% in the PHa@I-L, PHa@I-M, and PHa@I-H groups, respectively; after which, the release curves did not increase significantly. Because the residual drug concentration was low in the PBS after a certain period of release and the interference of the degradable polymer matrix of hydrogels, it is difficult to accurately measure the residual drug concentration after 10 days of release; that is why, only ∼78% of icariin was detected. In summary, these results can be regarded as the intermolecular interaction between Poloxamer 407 micelles and HA. Poloxamer 407 can form a self-assembled micelle structure in aqueous solution, and the micelles can be closely packed above the CGT (Cong Truc et al., 2011). In the PHa hydrogel, the high molecular weight with 1,000 kDa filled the space between the Poloxamer 407 micelles. Moreover, the HA molecules located between the micelles also serve as an inter-molecular crosslinker, which result in the formation of a highly dense inter-micellar structure when the temperature is higher than the CGT (Jung et al., 2017).

### Icariin-Loaded Hydrogels Promote Cell Proliferation and Viability

**FIGURE 2:**
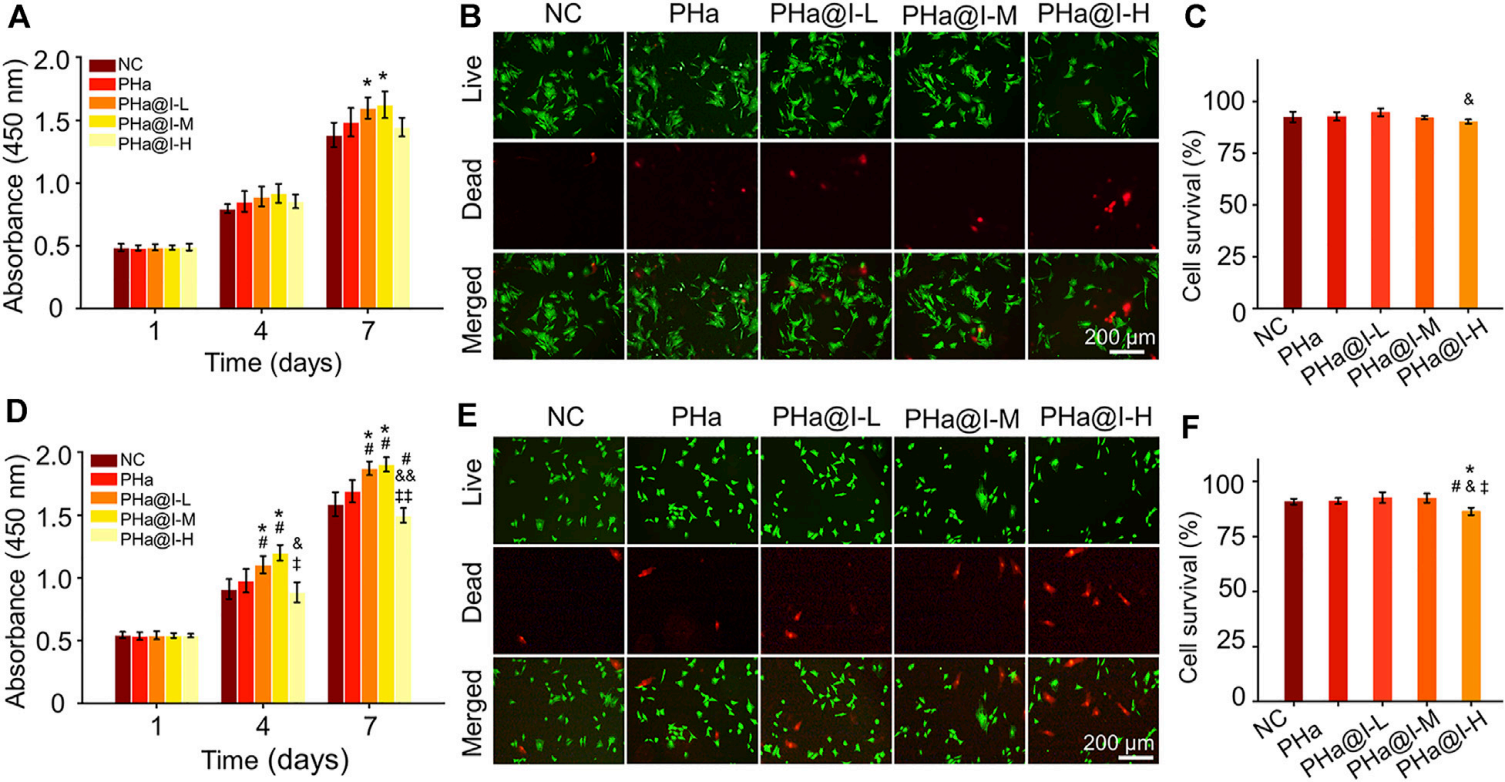
Biocompatibility of 3D cell culture system composed of icariin-loaded hydrogels on bone marrow mesenchymal stem cells (BMSCs) and chondrocytes. **(A)** BMSC proliferation at days 1, 4, and 7. **(B)** Calcein AM/PI staining of BMSCs at day 3. The green signal represents live cells, whereas the red signal represents dead cells. **(C)** Quantitative analysis of the survival rates of BMSCs according to Calcein AM/PI staining. **(D)** Chondrocyte proliferation at days 1, 4 and 7. **(E)** Calcein AM/PI staining of chondrocytes at day 3. The green signal represents live cells, whereas the red signal represents dead cells. **(F)** Quantitative analysis of the survival rates of chondrocytes according to Calcein AM/PI staining (*p < .05 compared with the NC group; ^#^ *p* < .05 compared with the PHa Group; ^&^ *p* < .05 and ^&&^ *p* < .01 compared with the PHa@I-L Group; ^‡^ *p* < .05 and ^‡‡^ *p* < .01 compared with the PHa@I-M Group, *n* = 3).

The biocompatibility of materials is a crucial issue for their biological applications. Herein, the effect of the 3D cell culture system, composed of icariin-loaded hydrogels on proliferation and viability of BMSCs and chondrocytes, was evaluated by CCK-8 analysis and Calcein AM/PI staining. As illustrated in Figure 2A, on the 7th day, the proliferation of BMSCs in the PHa@I-L Group and PHa@I-M Group was better than that in the NC Group (*p* < .05). Fluorescence images of Calcein-AM/PI staining showed that, although most BMSCs in each group were green-stained live cells, the PHa@I-H Group had the most red-stained dead cells (Figure 2B). Furthermore, the cell survival rates of BMSCs in the NC Group, PHa Group, PHa@I-L Group, PHa@I-M Group, and PHa@I-H Group were 92.54 ± 2.51%, 92.82 ± 1.95%, 94.77 ± 1.92%, 92.18 ± .90%, and 90.26 ± 1.04%, respectively (Figure 2C). For chondrocytes, the PHa@I-L Group and PHa@I-M Group can induce cell proliferation and maintain viability; however, the PHa@I-H Group indicated an inhibitory effect on proliferation and viability (Figures 2D–F). The introduction of HA and Poloxamer 407 into the hydrogel makes it an ECM component with excellent biocompatibility, which can produce a tendency to increase cell proliferation. Icariin is one of the effective ingredients of traditional Chinese medicine *Epimedium*. Appropriate concentration of icariin has a stimulatory effect on the proliferation of MSCs and chondrocytes (Ye et al., 2017; Liu et al., 2019; Wang et al., 2020). However, this inductive proliferation effect is related to the content of icariin in the hydrogels (Jiao et al., 2018). Icariin with proper concentration can promote cell proliferation, but too high concentration will cause drug toxicity, thereby inhibiting cell proliferation and activity (Fu et al., 2016). Several studies have also indicated that the icariin concentration above 10 μM was cytotoxic in a long period culture (Liu et al., 2010; Zhang et al., 2013; Liu et al., 2019). Our results demonstrated that the PHa hydrogel containing low and medium concentrations of icariin, namely, the PHa@I-L Group and PHa@I-M Group, showed a positive role in the proliferation and viability of BMSCs and chondrocytes. However, high concentrations of icariin in the hydrogel (the PHa@I-H Group) revealed an inhibitory effect on cell proliferation and viability.

### Icariin-Loaded Hydrogels Induce Bone Marrow Mesenchymal Stem Cells Chondrogenic Differentiation

In 2003, Murphy et al. (2003) reported that intra-articular injection of BMSCs alleviated the progress of OA in goat model, which aroused the interest of the people in the strategy of intra-articular injection of stem cells for the treatment of OA in the clinic. Since then, various studies have demonstrated that intra-articular injection of MSCs is helpful to ameliorate cartilage destruction and relieve pain in OA (Wang and Cao, 2014; Doyle et al., 2020; Kim et al., 2021). However, after the MSCs were injected into the OA joint cavity, only a few cells adhered to the cartilage defect, and their chondrogenic differentiation ability was not prominent and well controlled, which may limit their clinical applications (Koga et al., 2008; Chang et al., 2016; Ha et al., 2019). In addition to cell proliferation and survival, chondrogenic differentiation of stem cells is a crucial element for the treatment of OA. Therefore, we speculate that MSCs with more chondrogenic differentiation potential will contribute to the efficient repair of articular cartilage in OA. Herein, we encapsulated different amounts of icariin into the PHa hydrogels, attempting to screen an optimal hydrogel-drug formulation for inducing chondrogenic differentiation of BMSCs.

**FIGURE 3:**
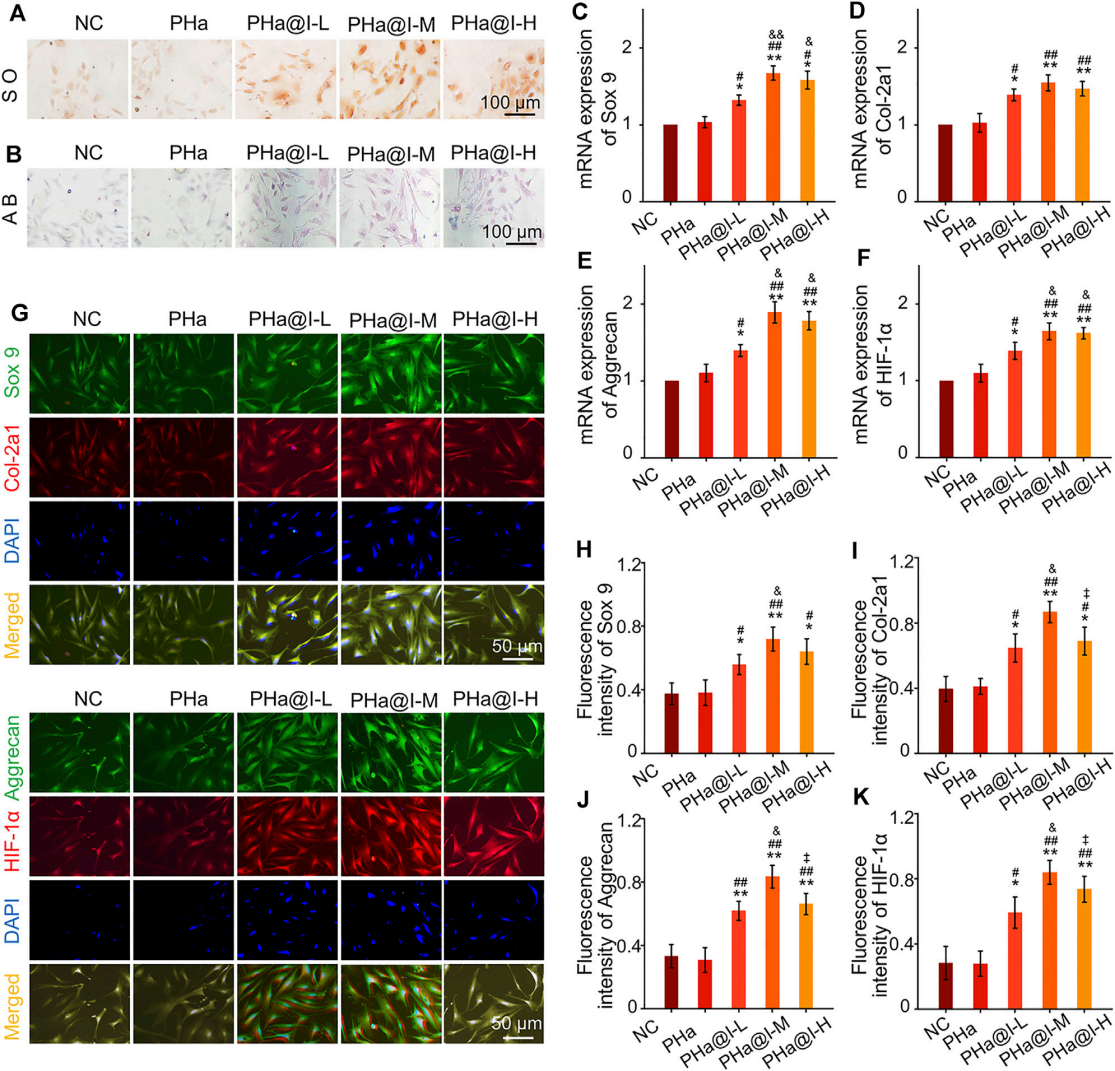
Icariin-loaded hydrogels as 3D cell culture systems induce BMSCs chondrogenic differentiation. **(A)** Safranin O staining of BMSCs. **(B)** Alcain blue staining of BMSCs. **(C–F)** RT-qPCR analysis of chondrogenic-related genes *Sox 9*, *Col-2a1*, *Aggrecan*, and *HIF-1α* in BMSCs after chondrogenic induction. **(G)** Double immunofluorescent staining of cartilage-specific markers Sox 9, Col-2a1, Aggrecan, and HIF-1*α* in BMSCs after chondrogenic induction. **(H–K)** Quantitative analysis of the immunofluorescence intensity of Sox 9, Col-2a1, Aggrecan, and HIF-1*α* (*p < .05 and **p < .01 compared with the NC group; ^#^ *p* < .05 and ^##^ *p* < .01 compared with the PHa Group; ^&^ *p* < .05 compared with the PHa@I-L Group; ^‡^ *p*< .05 compared with the PHa@I-M Group, *n* = 3).

Safranine O and Alcain blue staining were performed when BMSCs were cultured with chondrogenic induction medium in the 3D cell culture system for 12 days. As shown in Figures 3A, B, the results confirmed that icariin-loaded hydrogels could significantly induce chondrogenic differentiation of BMSCs, especially in the PHa@I-L Group and PHa@I-M Group. Furthermore, the results of RT-qPCR revealed that the expression of chondrogenic genes *Sox 9*, *Col-2a1*, *Aggrecan*, and *HIF-1α* were upregulated in groups of icariin-loaded hydrogels, which were significantly higher than that of the NC Group and PHa Group (Figures 3C–F). In addition, at the protein level, chondrogenic-related proteins including Sox 9, Col-2a1, Aggrecan, and HIF-1α were stained by double immunofluorescence (Figure 3G). As indicated in Figures 3H–K, quantitative analysis of the fluorescence intensity demonstrated that the expression of chondrogenic-related proteins in the groups of icariin-loaded hydrogels were significantly improved than those of the NC Group and PHa Group. It is worth mentioning that among the various icariin-loaded hydrogel groups, the expression of chondrogenic differentiation markers in the PHa@I-M Group was the most abundant, suggesting that this formula may be the optimal choice for intra-articular injection of cell-incorporating hydrogels to treat OA.

Previous research has demonstrated that icariin can upregulate the expression of cartilage-specific markers (e.g., Sox 9, Col-2a1, and aggrecan) and enhance the aggrecan production (Li et al., 2012). In addition, studies have revealed that icariin is an accelerator for ECM synthesis and can be regarded as a substitute for or cooperate with growth factors to directly enhance chondrogenic differentiation of BMSCs but not hypertrophy (Wang et al., 2014; Wang et al., 2018). Sox 9 is an early chondrogenic marker, which can promote the synthesis of collagen II and aggrecan. Herein, the results exhibited that icariin-loaded hydrogels notably upregulated the expression of the cartilage-specific genes, enhanced the fluorescent intensity and protein levels of Sox 9, Col-2a1, Aggrecan, and HIF-1*α*, and subsequently induced chondrogenesis of BMSCs. However, this chondrogenic induction effect is related to the content of icariin in the hydrogels. According to the above results, we consider that PHa@I-M hydrogel is the best candidate as a stimulator of chondrogenic differentiation of BMSC *in vivo* investigation.

### Icariin-Loaded Hydrogels Activate Wnt/*β*-Catenin Signaling

Wnt signaling is involved in multifarious cellular processes, such as cell proliferation, differentiation, apoptosis, and survival. The canonical Wnt pathway is commonly referred to as the Wnt/*β*-catenin signaling pathway, which mainly activates the particular gene expression in the nucleus (Fu et al., 2016). When Wnt proteins (e.g., Wnt3a) bind to their receptor, a series of downstream targets and cascade reactions are activated, such as the Dvl (an important target of Wnt signaling pathway) and GBP (a GSK-3*β* inhibition protein). Recently, Ling et al. (2009) indicate that the Wnt signaling plays a crucial role in the proliferation and survival of BMSCs. Wnt signaling pathway promotes proliferation, and inhibits adipogenesis and osteogenic differentiation of MSCs, which are related to the cytoplasm *β*-catenin accumulation and GSK-3*β* activity inhibition (Boland et al., 2004; Kennell and MacDougald, 2005). Then the accumulation of intracellular *β*-catenin is translocated into the nucleus to activate RNA transcription of downstream target genes.

**FIGURE 4:**
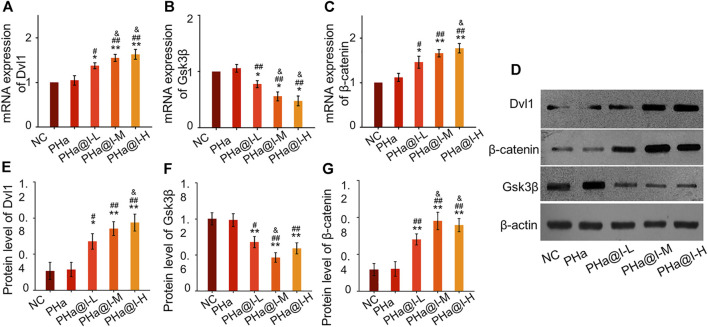
Icariin-loaded hydrogels as 3D cell culture systems activate Wnt/*β*-catenin signaling. **(A–C)** RT-qPCR analysis of Wnt/*β*-catenin signaling pathway-related genes *Dvl*, *GSK-3β*, and *β-catenin*. **(D–G)** Western blot analysis of Wnt/*β*-catenin signaling pathway-related proteins Dvl, GSK-3*β*, and *β*-catenin (*p < .05 and **p < .01 compared with the NC group; ^#^ *p* < .05 and ^##^ *p* < .01 compared with the PHa Group; ^&^ *p* < .05 compared with the PHa@I-L Group, *n* = 3).

Icariin-induced chondrogenic differentiation of MSCs has been widely used in cartilage tissue engineering, but the specific molecular mechanism is rarely reported. As shown in Figures 4A–C, RT-qPCR analysis indicated that the expression of *Dvl* and *β-catenin* were upregulated, and *GSK-3β* was inhibited after icariin-loaded hydrogel treatment in this study. In addition, Western blot results also demonstrated that these critical target proteins in the Wnt/*β*-catenin pathway changed accordingly, suggesting that the Wnt/*β*-catenin pathway was activated in the PHa@I-L Group, PHa@I-M Group, and PHa@I-H Group. Therefore, we speculate that the effects of 3D cell culture system composed of icariin-loaded hydrogels on promoting proliferation and chondrogenic differentiation of BMSCs may be related to the Wnt/*β*-catenin signaling pathway. Intra-articular injection of BMSCs incorporating PHa@I hydrogels reduces cartilage degeneration in OA.

**FIGURE 5:**
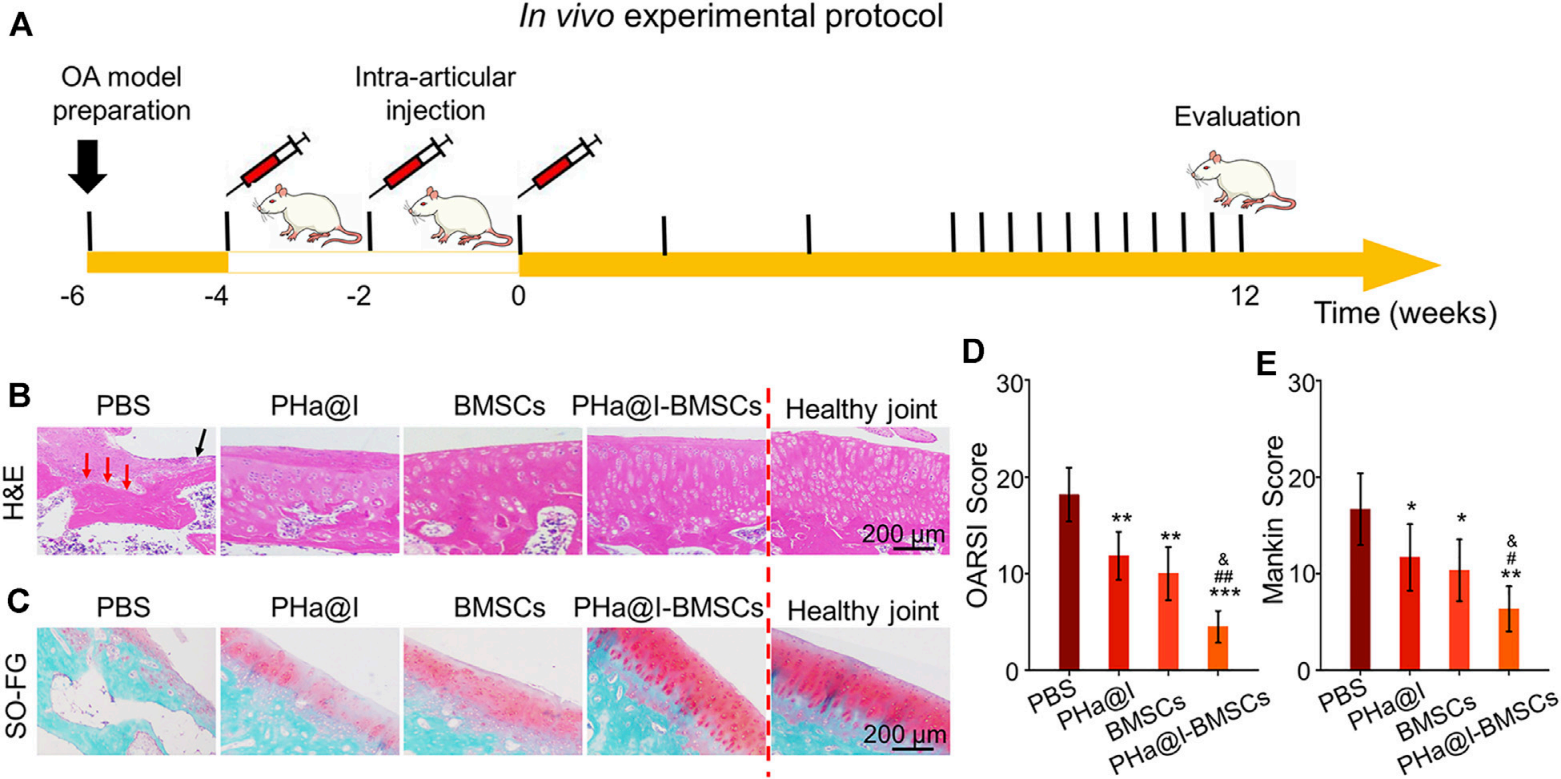
Intra-articular injection of BMSCs incorporating PHa@I hydrogels reduce cartilage degeneration in osteoarthritis (OA). **(A)** Timeline of *in vivo* experimental protocol. **(B)** Hematoxylin eosin (H&E) staining of articular cartilage at 12 weeks after intra-articular injection. **(C)** Safranin O-fast green staining of articular cartilage at 12 weeks after intra-articular injection. **(D)** Osteoarthritis Research Society International (OARSI) scores were statistically analyzed in each group. **(E)** Markin scores were statistically analyzed in each group (*p < .05, **p < .01, and ***p < .001 compared with the PBS group; ^#^ *p* < .05 and ^##^ *p* < .01 compared with the PHa@I Group; ^&^ *p* < .05 compared with the BMSC Group, *n* = 6).

OA is a chronic bone and joint disease, characterized by degeneration and deficiency of cartilage, osteophyte formation, resulting in joint pain, stiffness, and dysfunction, which is one of the major causes for the physical disability of the elderly. In this study, we developed an early nonsurgical treatment of OA by preparing an icariin-PHa hydrogel to carry BMSCs for intra-articular injection (Figure 5A).

After 12 weeks of injection, the articular cartilages were collected for histological evaluation. As displayed in Figure 5B, H&E staining of the weight-bearing part of the joints exhibited that in the PBS Group, articular cartilage missed and subchondral bone exposed (red arrow), as well as extensive necrotic cells (black arrow) were observed on the articular surface. Although the histological morphology of articular cartilage in the PHa@I Group and BMSC Group maintained a relatively complete gross shape, the cartilage layer became thinner and slightly matte, and the deep cells were vacuolated. The chondrocytes on the cartilage surface disappeared, and the deep chondrocytes were arranged irregularly and loosely. To our satisfaction, the articular cartilage was generally intact, and cells were regularly arranged in the PHa@I-BMSCs Group. In addition, safranin O-fast green staining was performed to observe the cartilage matrix. The results indicated that the integrity of the cartilage surface was destroyed in the PBS Group, leading to uneven distribution of cartilage matrix and slight or no staining. The PHa@I Group and BMSCs Group also presented insufficient staining of safranin O-fast green, indicating the loss of extracellular matrix, but it was better than that in the PBS Group. Conversely, the articular surface in the PHa@I-BMSCs Group indicated a good histological morphology and uniform matrix distribution (Figure 5C).

The OARSI cartilage histopathology assessment system and the Mankin scoring system were applied to estimate cartilage degeneration. In the PBS Group, PHa@I Group, BMSCs Group, and PHa@I-BMSCs Group, the scores of OARSI were 18.16 ± 2.78, 11.83 ± 2.48, 10.00 ± 2.75, and 4.50 ± 1.64 (Figure 5D), and the values of Mankin scoring were 8.33 ± 1.86, 5.83 ± 1.72, 5.16 ± 1.60, and 3.16 ± 1.16, respectively (Figure 5E). The scores in the PHa@I-BMSCs Group were significantly lower than those in the PBS Group, PHa@I Group, and BMSCs Group, suggesting that intra-articular injection of BMSCs incorporating PHa@I hydrogels could significantly reduce cartilage degeneration in OA. Intra-articular injection of BMSCs incorporating PHa@I hydrogels enhances chondrogenesis in OA.

**FIGURE 6:**
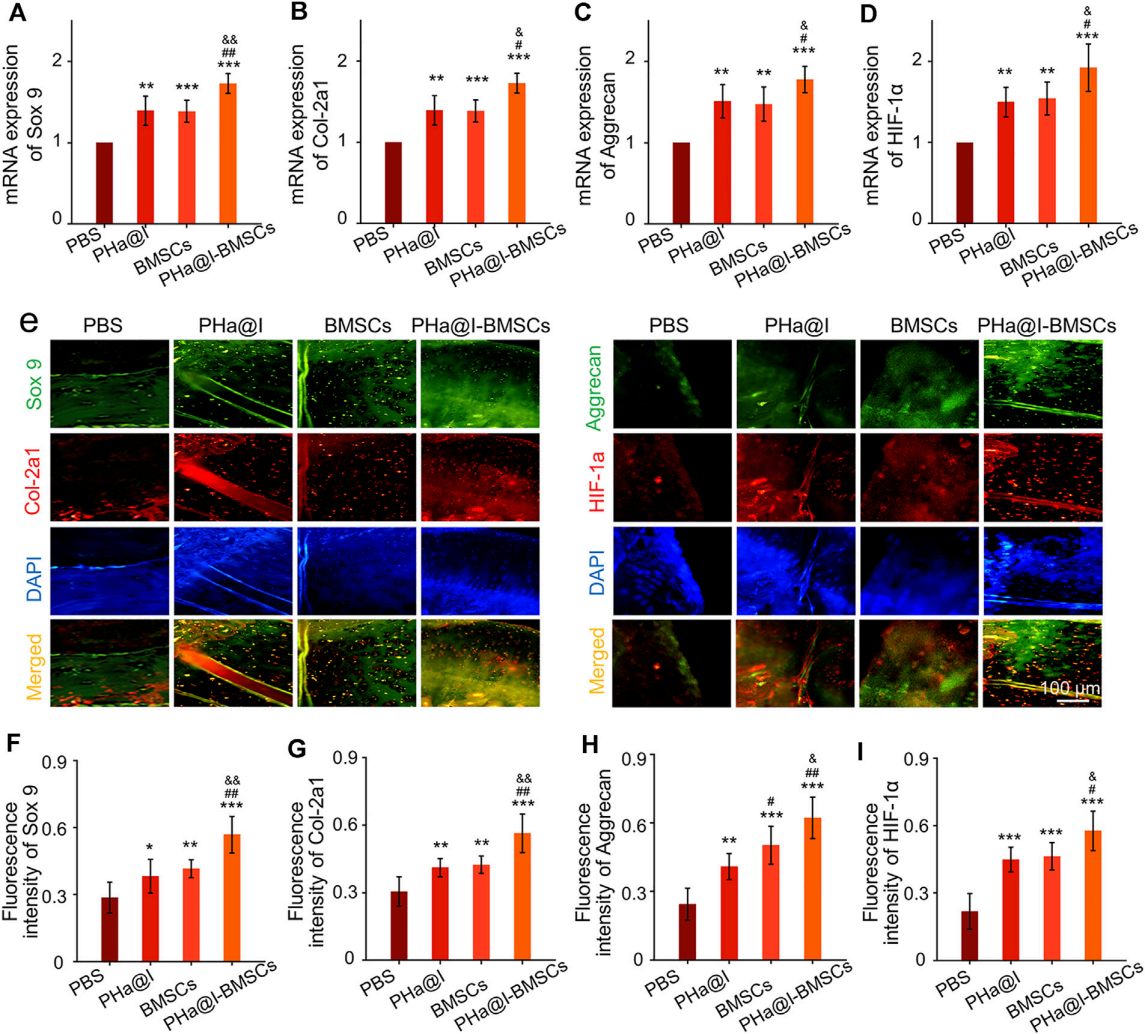
Intra-articular injection of BMSCs incorporating PHa@I hydrogels enhance chondrogenesis. **(A–D)** RT-qPCR analysis of chondrogenic-related genes *Sox 9*, *Col-2a1*, *Aggrecan*, and *HIF-1α* in cartilage at 12 weeks post-injection. **(E)** Representative double immunofluorescence images of Sox 9, Col-2a1, Aggrecan, and HIF-1*α* in cartilage at 12 weeks post-injection. **(F–I)** Quantitative analysis of fluorescence intensity of Sox 9, Col-2a1, Aggrecan, and HIF-1*α* in immunofluorescence staining (*p < .05, **p < .01, and ***p < .001 compared with the PBS Group; ^#^ *p*< .05 and ^##^ *p* < .01 compared with the PHa@I Group; ^&^ *p* < .05 compared with the BMSC Group, *n* = 6).

To further detect the expression of cartilage-related markers in the cartilage, RT-qPCR and immunofluorescence staining of the articular cartilage were performed to analyze the expression levels of Sox 9, Col-2a1, Aggrecan, and HIF-1*α* at 12 weeks post-injection. The expression of *Sox 9*, *Col-2a1*, *Aggrecan*, and *HIF-1α* at transcriptional level in PHa@I-BMSCs Group were significantly upregulated compared with the PBS Group, BMSC Group, and PHa@I Group. Moreover, the chondrogenic-related gene expression in the BMSC Group and PHa@I Group were also enhanced than that of the PBS Group (Figures 6A–D). Subsequently, we performed double immunofluorescence staining to further verify the expression of chondrogenic differentiation markers at the translation level (Figure 6E). Quantitative analysis was performed and indicated that the fluorescence intensity of Sox 9, Col-2a1, Aggrecan, and HIF-1*α*in each group had a similar trend with that at the gene level (Figures 6F–I). These results indicated that injection of icariin-loaded hydrogel or BMSCs alone can partially improve the expression of cartilage markers in arthritic cartilage, but the combination strategy can better promote chondrogenic differentiation of BMSCs encapsulated in the hydrogel.

**FIGURE 7:**
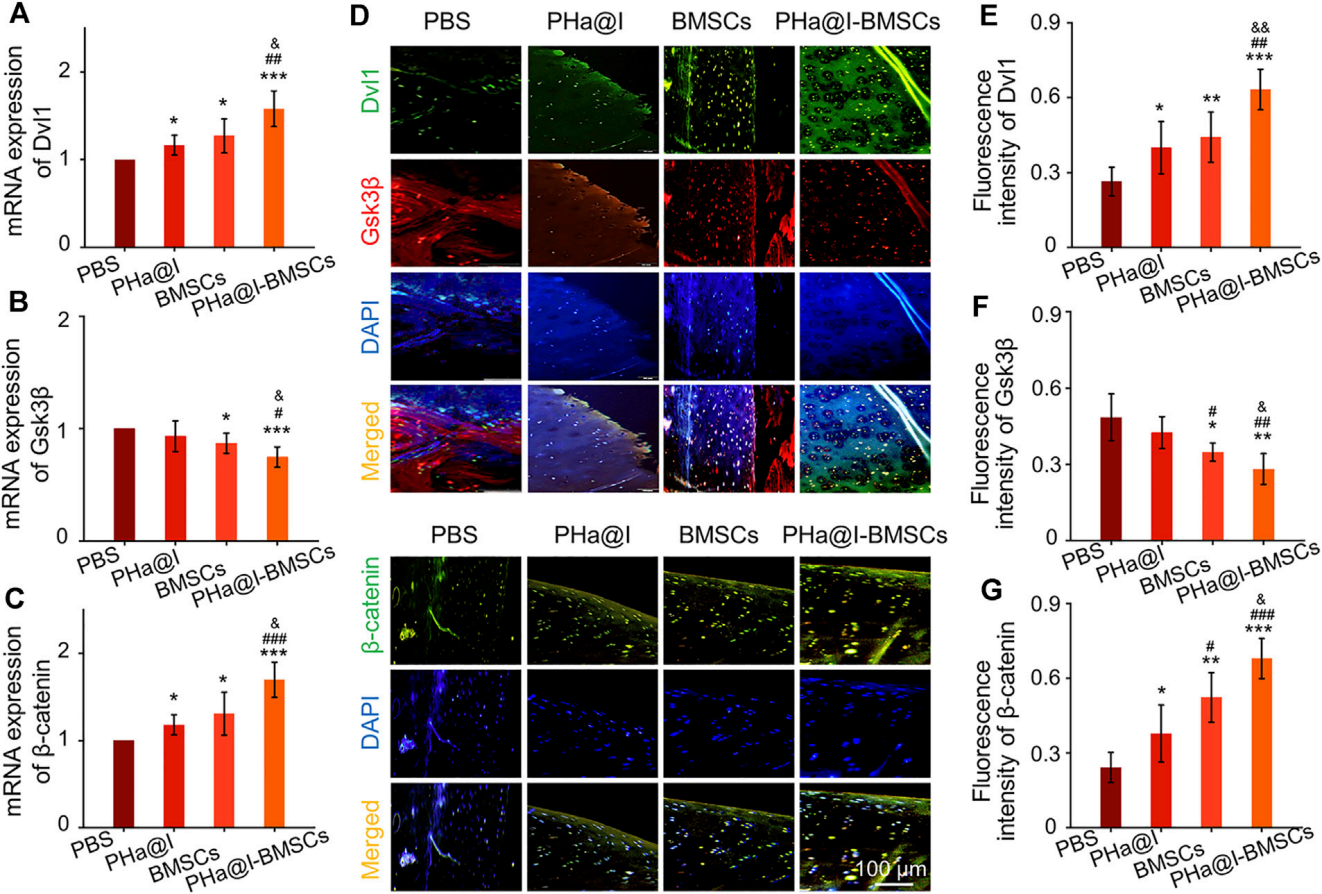
The Wnt/*β*-catenin signaling was significantly activated in the PHa@I-BMSCs Group. **(A–C)** RT-qPCR analysis Wnt/*β*-catenin signaling pathway-related genes *Dvl*, *GSK-3β*, and *β-catenin* in cartilage at 12 weeks post-injection. **(D)** Representative double immunofluorescence images of Dvl, GSK-3*β*, and *β*-catenin in cartilage at 12 weeks post-injection. **(E–G)** Quantitative analysis of fluorescence intensity of Dvl, GSK-3*β*, and *β*-catenin in immunofluorescence staining (*p < .05, **p < .01, and ***p < .001 compared with the PBS Group; ^#^ *p* < .05, ^##^ *p* < .01, and ^###^ *p* < .001 compared with the PHa@I Group; ^&^ *p*< .05 and ^&&^ *p* < .01 compared with the BMSC Group, *n* = 6).

Studies have revealed that icariin can induce cartilage regeneration and bone repair (Zeng et al., 2014; Yang et al., 2018). The main mechanism involved may be that icariin increases cartilage ECM synthesis and suppresses degradation, enhances cartilage-specific gene expression of chondrocytes, and promotes directed chondrogenesis of BMSCs (Sun et al., 2013; Wang et al., 2014; Wang et al., 2018). However, the safe concentration threshold of icariin was very low, and the cytotoxicity began to appear at the icariin concentration above 10–100 μM but with differences in the species from which the tested cells were obtained (Liu et al., 2010; Zhang et al., 2013; Jiao et al., 2018; Liu et al., 2019). Furthermore, the solubility of icariin is ∼25 μM at room temperature and much more at physiological temperature. Moreover, the solubility of icariin in different solutes is quite different. Icariin has poor solubility in water, but encapsulation in hydrogel can significantly improve its solubility through physical inclusion and hydrogen bonding (Xu et al., 2020). To reduce the cytotoxicity and maintain a long-term bioactivity of icariin in the articular cavity, the release profiles of icariin must be well controlled. In this study, we introduced a novel icariin-PHa hydrogel, in which icariin could sustain release for up to 12 days. Histological observation demonstrated that icariin-PHa hydrogel maintained its bioactivity for inducing chondrogenesis of BMSCs and promoting cartilage regeneration, thus, improving the histological morphology of OA. In addition, the specific molecular mechanism of chondrogenic differentiation was also investigated *in vivo*. RT-qPCR detection (Figures 7A–C) and immunofluorescence staining analysis (Figures 7D–G) indicated that the expression of *Dvl* and *β*-catenin were upregulated, GSK-3*β* was inhibited significantly after BMSC-incorporating icariin-loaded hydrogel injection, suggesting that the Wnt/*β*-catenin signaling was obviously activated in the PHa@I-BMSCs Group.

### Intra-Articular Injection of Bone Marrow Mesenchymal Stem Cells Incorporating PHa@I Hydrogels Inhibit Inflammation and Relieve Pain Caused by Osteoarthritis

**FIGURE 8:**
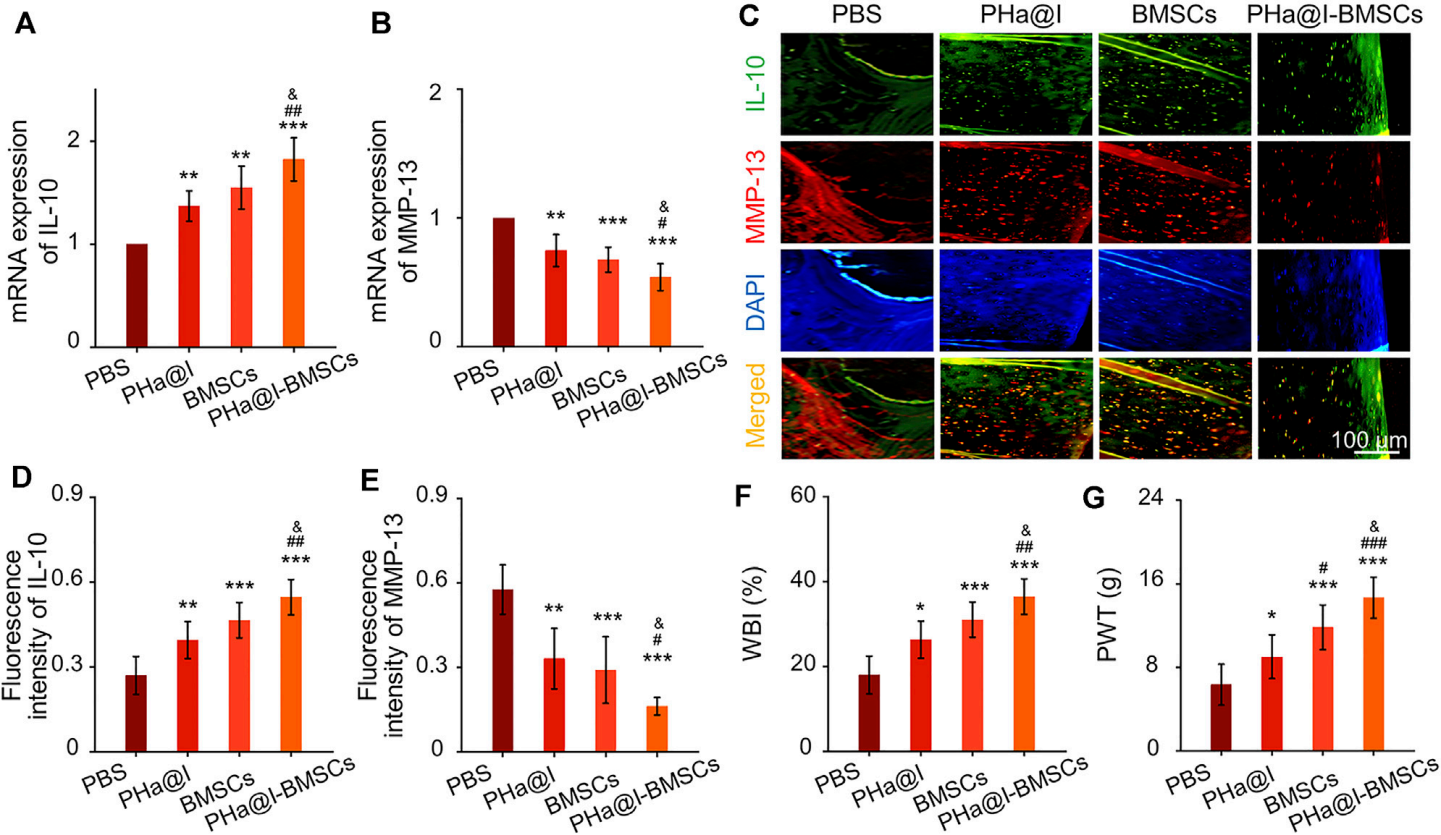
Intra-articular injection of BMSCs incorporating PHa@I hydrogels inhibit inflammation and relieve pain in OA. **(A,B)** RT-qPCR analysis *IL-10* and *MMP-13* in cartilage. **(C)** Representative double immunofluorescence images of IL-10 and MMP-13. (**D,E)** Quantitative analysis of fluorescence intensity of IL-10 and MMP-13 in immunofluorescence staining. **(F)** Weight-bearing capacity was calculated by weight-bearing index (WBI). **(G)** Paw withdrawal threshold (PWT) was measured to test mechanical allodynia (*p < .05, **p < .01, and ***p < .001 compared with the PBS Group; ^#^ *p* < .05, ^##^ *p* < .01, and ^###^ *p* < .001 compared with the PHa@I Group; ^&^ *p* < .05 compared with the BMSC Group, *n* = 3).

Cartilage degeneration is closely related to the main clinical symptoms of OA patients. It is widely known that inflammation response plays a critical role in OA pathogenesis due to it exacerbating pain and joint destruction. Proof from previous studies shows that interleukin-10 (IL-10) can alleviate pain symptoms and be used for pain relief therapy (Kwon et al., 2018). Proinflammatory cytokines and matrix metalloproteinases (MMP) show a critical function in the pathogenesis of OA (Kwon et al., 2018). Among them, MMP-13 shows an important role in cartilage remodeling and degradation due to its specificity in cutting Col-2. Previous studies have confirmed that excessive inflammatory reaction and high expression of MMP-13 can aggravate OA severity by inducing cartilage degradation (Lan et al., 2020; Hu and Ecker, 2021). In our study, the results indicated that the PHa@I-BMSCs obviously enhanced the expression of anti-inflammatory factor, *IL-10*, and inhibited the expression of proinflammatory marker, *MMP-13*, than the PBS Group, PHa@I Group, and BMSCs Group (Figures 8A,B). It is worth mentioning that the PHa@I Group and BMSC Group also had a certain role in regulating the local inflammatory microenvironment of OA, although it is not as obvious as the PHa@I-BMSCs Group. In addition, double immunofluorescence staining of IL-10 and MMP-13 proteins showed the same trend as RT-qPCR results **(** Figures 8C–E). Therefore, pure PHa@I hydrogel has certain anti-inflammatory effect due to the presence of hyaluronic acid. The immunomodulation and anti-inflammatory effects of BMSCs are amplified in OA after being encapsulated by PHa@I hydrogel (Han et al., 2015; Ye et al., 2019; Yu et al., 2021).

Peripheral pain mechanisms are related to the direct activation of nociceptors and sensitization of nociceptors caused by the inflammation reaction in the articular cavity (Meeus et al., 2012; Boyden et al., 2016). Responding to the inflammatory mediators, intracellular signaling pathways cause a phosphorylation cascade, which decreases the threshold for nociceptor neurons to activate action potentials, eventually leading to increased pain sensitivity (Pinho-Ribeiro et al., 2017). Quantitatively, the WBI values of the PBS Group, PHa@I Group, BMSCs Group, and PHa@I-BMSCs Group were 34.87 ± 1.98%, 38.76 ± 2.31%, 40.59 ± 2.24%, and 45.13 ± 3.11%, respectively (Figure 8F). The WBI below 50% means a decreased weight-bearing of the treated leg and increased contribution of the contralateral leg in bearing weight. The WBI reduces in the limb of the inflamed joint due to the weight bearing aggravating the sensation of pain, a major symptom of OA (Chen et al., 2020). In addition, the PWT of the PBS-treated rats decreased to 6.33 ± 1.96 g, which was significantly lower compared with the PHa@I Group, BMSCs Group, and PHa@I-BMSCs Group. With the combination of icariin-loaded hydrogel and BMSC injection, the PHa@I-BMSCs Group significantly reduced OA-induced hypersensitivity compared with the PHa@I Group and BMSC Group (Figure 8G). Herein, therapeutic effects of pain relief after intra-articular administration may be owing to the effective anti-inflammatory capacities of the icariin-loaded hydrogel and BMSCs in OA joint cavity.

## Conclusion

In this study, a novel intra-articular strategy, namely, injection of the BMSCs incorporating icariin-loaded thermosensitive hydrogel, was designed for the treatment of DMM-induced OA. Stable release of bioactive icariin from hydrogel can induce BMSC proliferation and accelerate chondrogenic differentiation through Wnt/*β*-catenin signaling pathway. Notably, intra-articular injection of this icariin-loaded hydrogel-encapsulated BMSCs provides an efficient outcome of retarding cartilage destruction, regulating inflammatory microenvironment, and relieving pain, suggesting a great prospect in the remission of OA. However, although surgical destabilization of the medial meniscus is a classic method to prepare animal models of OA, it cannot fully simulate all the pathological characteristics of degenerative changes in the joints in the elderly. The development of gene-editing animal models may be a potential strategy to solve this problem. In addition, our hydrogel has a stable and continuous rhythm for the release of drugs and lacks the induction of the microenvironment to regulate the release rate. The preparation and development of hydrogels that are intelligently responsive to the disease microenvironment may provide an individualized release curve in response to the severity of OA, thereby providing a customized treatment strategy.

## Data Availability

The original contributions presented in the study are included in the article/supplementary material. Further inquiries can be directed to the corresponding author.
